# Tilted Implants for Full-Arch Rehabilitations in Completely Edentulous Maxilla: A Retrospective Study

**DOI:** 10.1155/2012/180379

**Published:** 2012-10-23

**Authors:** Nicolò Cavalli, Bruno Barbaro, Davide Spasari, Francesco Azzola, Alberto Ciatti, Luca Francetti

**Affiliations:** Department of Biomedical, Surgical and Dental Sciences, Oral Implantology Research Center, Università degli Studi di Milano, IRCCS Istituto Ortopedico Galeazzi, 20161 Milan, Italy

## Abstract

*Purpose*. The aims of this study were to assess the treatment outcome of immediately loaded full-arch fixed bridges anchored to both tilted and axially placed implants in the edentulous maxilla and to evaluate the incidence of biological and prosthetic complications. *Materials and Methods*. Thirty-four patients (18 women and 16 men) were included in the study. Each patient received a maxillary full-arch fixed bridge supported by two axial implants and two distal tilted implants. A total of 136 implants were inserted. Loading was applied within 48 hours of surgery and definitive restorations were placed 4 to 6 months later. Patients were scheduled for followup at 6, 12, 18, and 24 months and annually up to 5 years. At each followup plaque level and bleeding scores were assessed and every complication was recorded. *Results*. The overall follow-up range was 12 to 73 months (mean 38.8 months). No implant failures were recorded to date, leading to a cumulative implant survival rate of 100%. Biological complications were recorded such as alveolar mucositis (11.8% patients), peri-implantitis (5.9% patients), and temporomandibular joint pain (5.9% patients). The most common prosthetic complications were the fracture or detachment of one or multiple acrylic teeth in both the temporary (20.6% patients) and definitive (17.7% patients) prosthesis and the minor acrylic fractures in the temporary (14.7% patients) and definitive (2.9% patients) prosthesis. Hygienic complications occurred in 38.2% patients. No patients' dissatisfactions were recorded. *Conclusions*. The high cumulative implant survival rate indicates that this technique could be considered a viable treatment option. An effective recall program is important to early intercept and correct prosthetic and biologic complications in order to avoid implant and prosthetic failures.

## 1. Introduction

Several long-term prospective and retrospective studies reported high survival and success rates for implant-supported prosthesis for full-arch rehabilitations of atrophic jaws [1–3]. The described full-arch rehabilitations were supported by implants placed in the median region of jaws, between the two mental foramina in the mandible and between the mesial walls of maxillary sinus. They supported a full prosthesis with distal cantilevers.

In the atrophic maxilla, even though sinus augmentation procedures were described as effective in creating conditions for implant placement [4, 5], the occurrence of several complications was reported in the literature [6].

Tilted implants were suggested to be useful in the treatment of edentulous jaws avoiding the bone augmentation procedures and the involvement of anatomical structures during surgery [7]. Furthermore, tilting of distal implants in full-arch rehabilitation allows to reduce cantilever length and to augment the anteroposterior distance between the most anterior implant emergence and the most posterior ones with several prosthetic advantages [8, 9]. 

The All-on-Four surgical and prosthetic procedure was proposed to rehabilitate edentulous arches without any bone augmentation procedures, using distal tilted implants to obtain prosthetic and surgical advantages as described before [10, 11]. Tilted implants should be placed mesially or in direct contact with the mesial walls of the maxillary sinus, without invasion or rupture of the Schneiderian membrane [12].

This procedure was validated by scientific literature in terms of implant success of survival both in short and in medium term, demonstrating that the use of tilted implants was not related to an increased bone resorption [9, 11, 13, 14].

The aim of this retrospective study was to investigate and present data about prosthetic and biological complications occurred in patients treated with full-arch maxillary rehabilitations supported by a combination of tilted and upright implants. Also implant survival rates were discussed and retrieved from clinical databases.

## 2. Materials and Methods

The Inclusion Criteria were as follows.

1. 18 years or older of any race and gender.
2. Patients in general good health condition, able to undergo surgical treatment and restorative procedures (ASA-1/ASA-2).
3. Completely edentulous maxilla or presence of teeth with an unfavorable long-term prognosis.
4. Adequate bone height and thickness in the region between the first premolars for the placement of implants at least 10 mm long and 4 mm wide.
5. Presence of extremely resorbed maxilla that would have needed bone augmentation for placing implants in a region posterior to the first premolars.
6. Patients who refused any kind of bone augmentation procedure.

The Exclusion Criteria were as follows.

1. Presence of acute infection at the implant site; hematologic diseases; serious problems of coagulation; diseases of the immune system; uncontrolled diabetes; metabolic diseases affecting bone; pregnancy or lactation.
2. Inadequate oral hygiene level (full-mouth plaque score and full-mouth bleeding score greater than 20%) and poor motivation to maintain good oral hygiene throughout the study.
3. Irradiation of the head or neck region or chemo-therapy within the past 60 months.
4. Severe bruxism or clenching.

Participants were informed about the nature of the study and signed an informed consent.

Preliminary screening was performed using a careful clinical examination of the patient, panoramic orthopantomographs, computerized tomographic scans, accurate blood tests, electrocardiography, and cardiological examination. All included patients were scheduled to be followed for up to 6 years after loading. 

### 2.1. Surgical Protocol

Patients received the following presurgical prophylactic drug therapy:

- antibiotics, amoxicillin and clavulanic acid 2 g 1 hour before surgery,
- chlorhexidine digluconate 0.2% mouthwash starting 3 days before surgery.

All surgeries were performed under local anesthesia with articaine chlorohydrate with adrenaline 1 : 100,000 and intravenous sedation with diazepam.

A crestal incision was made starting in the first molar position. All hopeless teeth, if present, were extracted and sockets were carefully debrided. Where necessary, a regularization of the edentulous bone ridge was performed with rotating instruments and/or bone forceps. Each patient received four implants (Brånemark System MKIV or NobelSpeedy Groovy, Nobel Biocare AB, Goteborg, Sweden) according to a previously described protocol (All-on-Four, Nobel Biocare AB, Göteborg, Sweden), with the the two distal implants tilted by approximately 30 degrees with respect to the occlusal plane and the two anterior implants axially inserted. To allow an immediate rehabilitation, each implant was inserted with a final torque of 40 to 50 Ncm. Multi-Unit Abutments (MUA, Nobel Biocare AB) were connected to the implants. On distal implants, abutments angulated 17 or 30 degrees with respect to the long axis of the fixture were positioned to obtain an optimal orientation for the prosthetic screw access, while straight abutments were placed over the anterior implants. An impression was taken utilizing a silicon putty polyvinlsiloxane directly on the coping. Then, four healing caps were placed upon the multiunit abutments.

Patients were discharged with the following postsurgical drug therapy:

- antibiotics, amoxicillin and clavulanic acid 1 g every 12 hours for six days after surgery;
- analgesics, naproxen sodium 550 mg for the first three days from surgery;
- chlorhexidine digluconate 0.2% mouthwash for 7 days following surgery.

### 2.2. Prosthetic Phase

**Figure 1:**
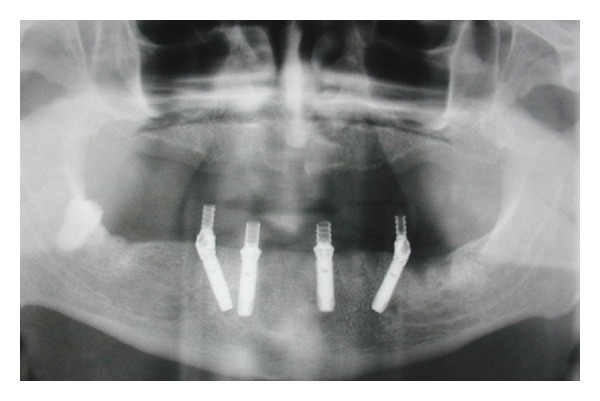
Pretreatment orthopantomography.

**Figure 2:**
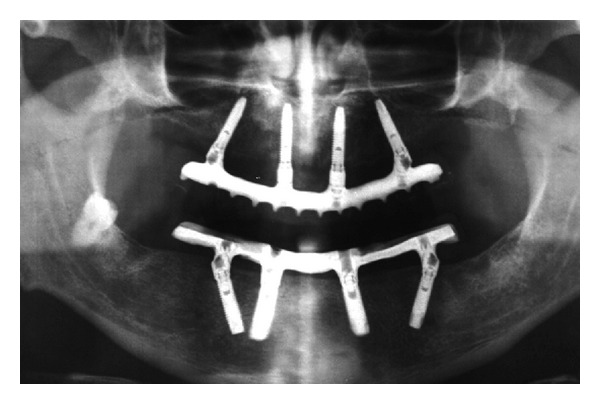
One year posttreatment orthopantomography.

**Figure 3:**
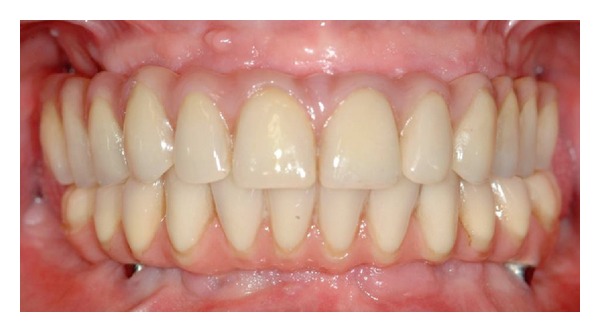
Frontal view of the definitive prosthesis.

**Figure 4:**
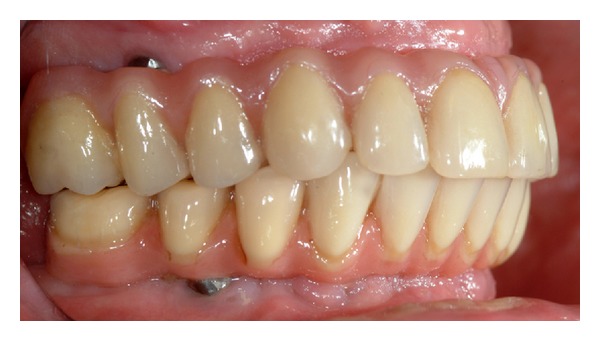
Lateral view of the definitive prosthesis.

**Figure 5:**
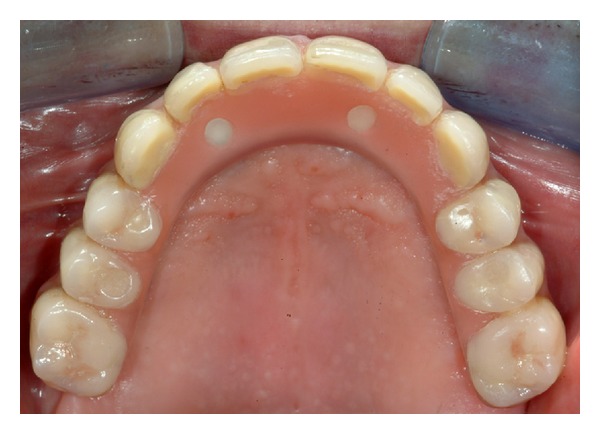
Occlusal view of the definitive prosthesis.

Within 48 h from surgery an acrylic temporary prostheses with 10 teeth and no cantilever was placed over the abutments. Screws were tightened over the MUA with a torque of 10 Ncm, following the manufacturer's instructions (Figure 1). All centric and lateral contacts were assessed by a 40 mm articulating paper and adjusted if necessary until they were present only between 33 and 43, according to the Maló protocol [10]. The screw access was then covered with provisional resin cement. After 6 months of loading, in the absence of pain and inflammatory signs, the patients received the final prosthesis (Figure 2). The defenitive prosthesis was composed by a titanium framework fabricated by means of the CAD-CAM Procera system (Nobel Biocare AB), acrylic pink resin, and composite resin teeth (Figures 3, 4, and 5).

### 2.3. Followup and Data Collection

The patients were scheduled for weekly control visits during the first month after surgery. During each visit, prosthetic functionality and tissue healing were evaluated. Every 3 months, oral hygiene level was evaluated. After defenitive prosthesis delivery patients were scheduled for follow-up visit every 6 months for the first two years and yearly thereafter up to 6 years.

At each follow-up visit, mobility of the prosthetic structure and occlusion was checked, any prosthetic or biological complication was recorded, plaque level and bleeding score was assessed, and periapical radiographs using a paralleling technique and an individual X-ray holder were performed for evaluation of peri-implant bone level change over time. 

## 3. Results 

From April 2007 to April 2011, a total of 34 healthy patients (18 women and 16 men; mean age 58.7 years; range 44 to 84 years) were rehabilitated with an immediately loaded implant-supported fixed maxillary prosthesis supported by four implants. 19 patients were smokers (average daily consumption: 16.3 cigarettes per day), with 8 of them smoking 20 cigarettes per day or more.

A total of 136 implants were inserted (implants' length ranges from 10 mm to 15 mm; mean lenght 12.2 mm), of whom 68 with an axial inclination and 68 tilted by 30°. All implants had a diameter of 4 mm. All patients received the provisional prosthesis as planned within 48 hours of surgery. The follow-up range was from 12 to 73 months (mean 38.8 months).

Up to date no implant failures were recorded, so the cumulative implant survival rate was 100% (Table 1).

**Figure 6:**
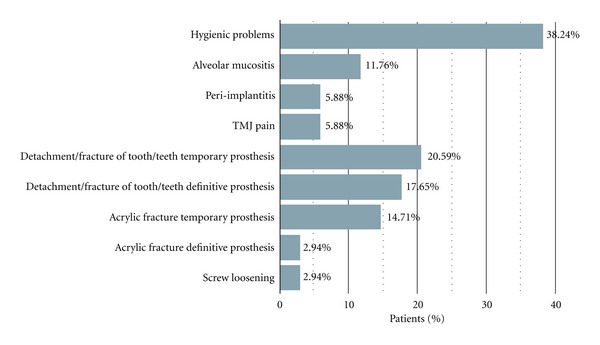
Graphical representation of the patient-related complication incidence.

Complication incidence over time was showed in Table 2 and in Figure 6.

Biological complications were documented consisting in alveolar mucositis in 4 patients (11.76% patients), peri-implantitis in 2 patients (5.88% patients), and temporomandibular joint (TMJ) pain in 2 patients (5.88% patients). Both TMJ pain cases were solved after the adjustment of centric and lateral contacts. 

The most common prosthetic complication was the fracture or detachment of one or more resin teeth that occurred in 10 patients (29.41% patients). In 7 patients it took place in the temporary prosthesis (20.59% patients) while in 6 patients in the definitive one (17.65% patients), in 3 of them happened in both. Minor acrylic resin fractures of the temporary prosthesis occurred in 5 patients (14.72% patients) and in 1 of them also in the definitive prosthesis (2.94% patients). 

Prosthetic screw loosening was recorded in one patient (2.94% patients). Twenty-one patients had no prosthetic complications (61.7% patients).

Hygienic problems were recorded in 13 patients (38.24% patients), but in most cases the patient was motivated to a better oral hygiene and the problem was solved without developing in alveolar mucositis or peri-implantitis. No patients' dissatisfaction was recorded.

## 4. Discussion

In this study medium-term data about implant and prosthetic complications were reported from a cohort of patients treated following the All-on-Four protocol. All implants were functioning determining the 100% cumulative survival rate. However, some prosthetic or hygienic complication occurred in a relatively high number of patients (almost 30%).

In clinical records the most reported parameter to evaluate the effectiveness of an implant-supported rehabilitation is the survival rate, meaning whether the implant is still physically in the mouth or has been removed. 

The commonly accepted criteria for the assessment of implant success were proposed by Albrektsson et al. [15]. 

Misch et al. in a consensus conference in 2007 [16] assessed as success parameters no pain in function, absence of observed clinical mobility, radiographic bone loss from surgery lower than 2 mm, and no exudates history. 

In the present study the patient-related implant survival rated is 100%, while the patient-related implant success rate results were 94.22% because implants with peri-implantitis cannot be considered successful.

However those parameters seem no longer sufficient to assess the clinical efficiency of current implant prosthetic methodologies [17].

A number of studies reported implant survival rates for this type of rehabilitation in edentulous maxillas. 

Recently some authors reported 98.96% of implant survival rate after 3 years from loading for 24 maxillary rehabilitations without any prosthetic complete failure [18]. 

Other authors reported good performances of this technique, in terms of implant survival rate and function in a large cohort of 276 patients, evaluated after 16 months from prosthesis placement [19].

A retrospective investigation performed by Babbush and coworkers described a 99.3% of implant survival rate for edentulous maxillas rehabilitated through the All-on-Four technique for up to 29 months of loading [14]. Also in this study the final prosthesis survival rate was 100%.

Another retrospective study, published by Malo et al. in 2011, reported data about 242 patients treated with a combination of two tilted and two upright implants [11]. Nineteen implants were lost in 17 patients, with a 5-year survival rate estimation of 93% and 98% at patient and implant level, respectively. Prosthesis survival rate was 100%. 

Even though scientific literature reported, high survival rates for implants and prosthesis used in this type of rehabilitation, there is a lack of description of minor prosthetic and implant complications that may occur.

A recent review of the literature about rehabilitation of atrophic maxilla with tilted implants reported implant success rates varying from 91.3% to 100% for 666 axial implants and 92.1% to 100% for 782 tilted ones evaluating 319 patients [20]. Only few minor prosthetic complications were reported but there is a lack of description of such occurrence.

Fischer and Stenber reported a description of long-term complication for full-arch maxillary prosthesis supported by upright implants [21, 22]. No abutment or screw fractures were reported. Up to 82% of prosthesis experienced complications in the 10-year follow-up period, and the most common complication was tooth fracture (4.7 resin-related complications per prosthesis). Only 4% of metal frameworks fractured and 9% were remade after 10 years.

Other report on a large cohort of patients with mandibular rehabilitations reported that resin or veneer fractures were the most frequent complication after 15-year followup [23]. The same results were reported for maxillary restorations [24].

Considering prosthetic complications, other authors reported that the most common complications were prosthetic tooth fracture, tooth wear, maxillary hard relines, and screw complications in cases of mandibular restorations [25].

Also in the present study the most common prosthetic complication was the detachment of teeth, especially in the provisional restoration. In final restorations some resin-related complications were reported too. Such occurrences were easily solved within one week and did not cause major complications at implant level. 

Hygienic complications were considered in the present study, because an early diagnosis of a problem in maintaining dental implant soft tissue health is necessary to reduce the prevalence of peri-implant diseases [26]. 

It has to be considered that the prevalence of peri-implant inflammatory disease has described to have a prevalence ranging from 50% to 90% of implants considering peri-implant mucositis (8–10 years) and from 12% to 43% of implants considering peri-implantitis (9–11 years) [27], and so, a strict control of hygienic problems is mandatory in the long-term maintenance.

Another observation deriving from the results of the present report is that despite the relatively high rate of minor prosthetic or hygienic complication, all implants survived and no failures were reported. This confirmed that an effective recall program is important to individuate complications in the beginning avoiding the evolution of these in major complication that may lead to implant failure.

In conclusion, the present study showed that the use of angled implants to rehabilitate atrophic maxillas could be a viable alternative to bone augmentation procedures in the posterior area and allowed a good functional and aesthetic patients' satisfaction.

Prosthetic and biologic complication should be early intercepted and corrected to avoid implant and prosthetic failures.

## Figures and Tables

**Table 1:** Cumulative survival rate.

Interval	Number of implants	Failed	CSR%
0–6 mo	136	0	100
6–12 mo	136	0	100
12–18 mo	132	0	100
18–24 mo	108	0	100
24–36 mo	108	0	100
36–48 mo	80	0	100
48–60 mo	36	0	100
60–72 mo	16	0	100

**Table 2:** Complication incidence over time.

Hygienic problems	38,24%
Al. mucositis	11,76%
Peri-implantitis	5,88%
TMJ pain	5,88%
Detachment/fracture of tooth/teeth in temporary prosthesis	20,59%
Detachment/fracture of tooth/teeth in definitive prosthesis	17,65%
Acrylic fracture in temporary prosthesis	14,71%
Acrylic fracture in definitive prosthesis	2,94%
Screw loosening	2,94%
